# Appendicitis mimicry of dolichocolon

**DOI:** 10.1093/jscr/rjad565

**Published:** 2023-10-17

**Authors:** Kayla K Brown, Mercedes C Jolley, Dean A Kocay

**Affiliations:** Graduate Medical Education, General Surgery Residency Program, St. David’s South Austin Medical Center, Austin, TX 78704, United States; Graduate Medical Education, General Surgery Residency Program, St. David’s South Austin Medical Center, Austin, TX 78704, United States; St. David’s South Austin Medical Center, Austin, TX 78704, United States

**Keywords:** dolichocolon, appendicitis, chronic constipation, general surgery, pediatric surgery, colorectal surgery

## Abstract

Dolichocolon is an under-reported, under-diagnosed etiology of chronic constipation that is often overlooked as being a primary diagnosis. We present a case of an undiagnosed dolichocolon in a young adult female whose initial presentation was concerning of appendicitis. Eventually, the patient underwent a subtotal colectomy as a definitive treatment for chronic constipation. Dolichocolon is an anatomical variant that can have severe lifelong consequences, such as chronic constipation, which greatly affects a patient’s quality of life and overall health if undiagnosed. The purpose of this case report is to raise awareness among the surgical community regarding the significance of early dolichocolon diagnosis, prevent misdiagnosis, and ultimately improve patient outcomes, including reducing complications associated with chronic constipation.

## Introduction

Approximately 14%–16% of the population in developed countries struggle with chronic constipation, with symptoms to include nonspecific, generalized symptoms including abdominal pain, weight fluctuation, and indigestion [1]. Furthermore, the etiology of chronic constipation is even vaster than its nonspecific symptoms, one being dolichocolon, with an incidence rate of 1.9%–28.5% [1, 2].

Dolichos, or δολιχός, is Greek for ‘long’. Although the definition of dolichocolon has been described as early as 1820 and named in 1914, it is not a common consideration for chronic constipation and is often overlooked in pediatric and young adult populations [3]. Only recently has dolichocolon been recognized as a distinct radiographic diagnosis, as it is generally described as ‘an elongation or redundancy of the colon with loop formation’ [4]. It is believed to be an inherent anatomical variant closely linked to chronic constipation [5]. This condition can be most effectively diagnosed radiographically with a colon transit study and barium enema.

Dolichocolon can be remedied by resecting the redundant portion and restoring it to its normal physiologic length and position. Nonetheless, colon resection carries risks including anastomotic complications, surgical site infection, bowel dysfunction, sexual dysfunction, and anastomotic stricture [6]. Numerous articles have reported positive outcomes for those with dolichocolon, with one study citing a success rate of 90% and improvements in abdominal pain and bloating, increased bowel movement frequency, and improved quality of life [7, 8].

## Case report

**Figure 1:**
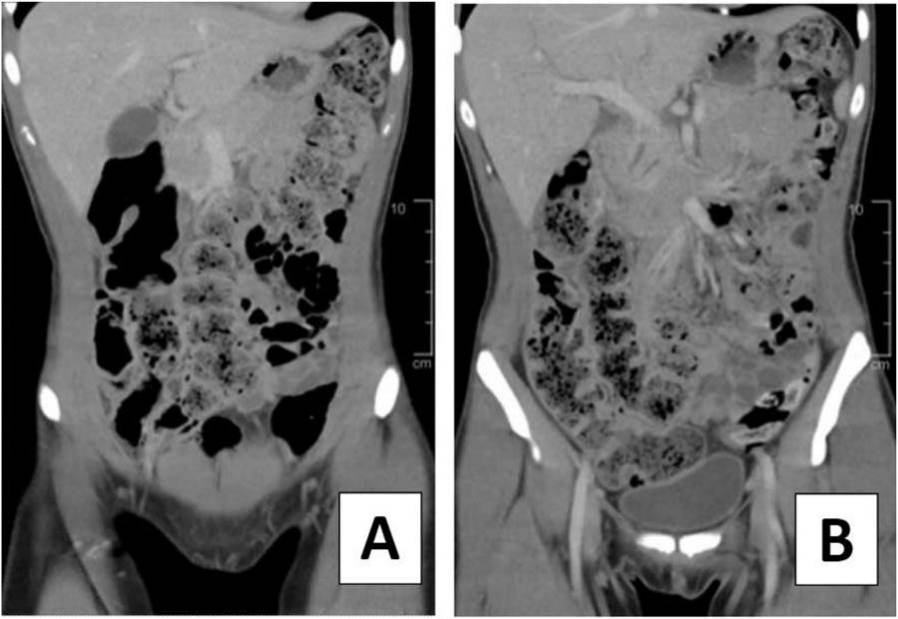
CT scan of 23-year-old female on hospital Day 1. (A, B) Coronal views depicting moderate to large fecal load.

A 23-year-old female presented to the emergency department with right lower quadrant abdominal pain. She also experienced nausea, fever, chills, and malaise. Additionally, she reported a history of chronic constipation, with her last bowel movement occurring several days prior to her hospital visit. On physical examination, tenderness was noted at McBurney’s point and a positive Rovsing sign was observed. Patient had a leukocytosis of 14 000 and an abdominal computed tomography (CT) with intravenous contrast was significant for moderate to large proximal fecal load and a normal-appearing appendix (Fig. 1).

The patient underwent a laparoscopic appendectomy on hospital Day 2. Exploration of the abdominal cavity revealed a retrocecal normal-appearing appendix. Further exploration of the abdominal cavity was completed, noting a significantly dilated and chronically adhered sigmoid and proximal ascending colon and a dilated and tortuous transverse colon that descended into the inferior abdomen and pelvis. Postoperatively, she was discharged from the hospital, with instructions to follow up as an outpatient to investigate dolichocolon as a probable source of her chronic constipation and abdominal pain.

**Figure 2:**
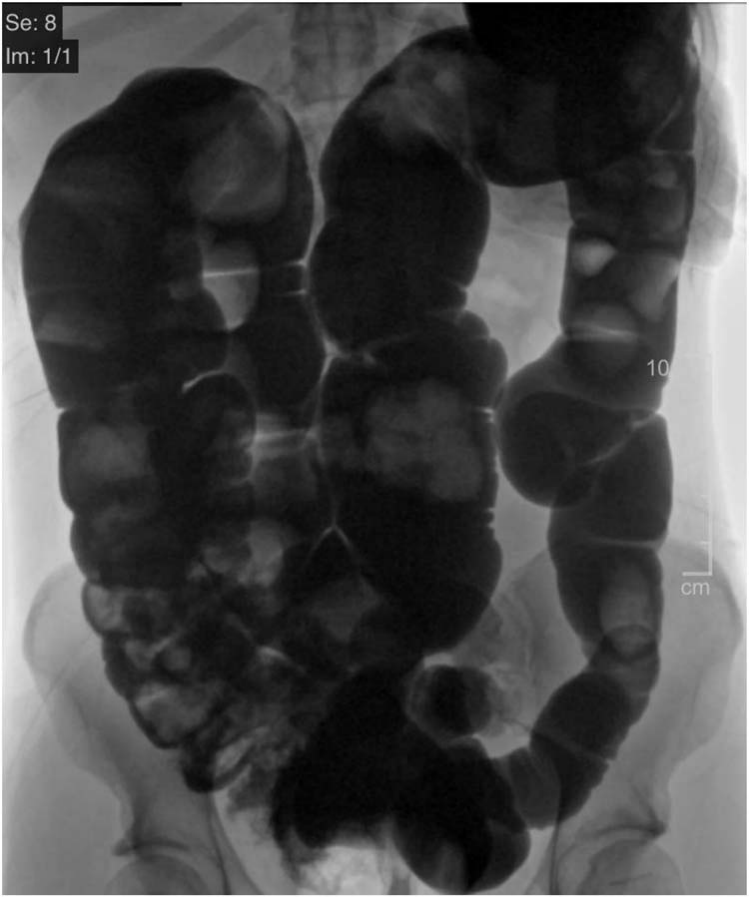
CT Diatrizoate meglumine (DM) enema with air contrast. DM flows in a retrograde manner from the rectum to the cecum without evidence of obstruction or mass.

**Figure 3:**
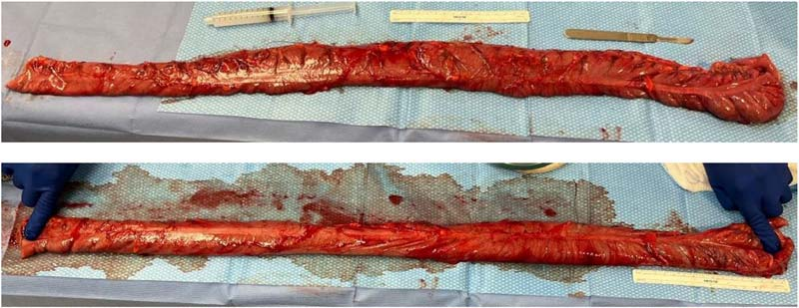
Intraoperative findings from hand-assisted laparoscopic extended right colectomy/subtotal colectomy with anastomosis. (Top) 80 cm (2.62 ft) resected segment of the dilated redundant ascending colon. (Bottom) 95 cm (3.12 ft) resected segment, on stretch.

Two weeks following discharge, the patient completed a mechanical bowel prep and was admitted electively to undergo a colonoscopy and a laparoscopic extended right colectomy with anastomosis. Preoperative CT imaging depicted significant redundancy of the large intestine without evidence of obstruction or mass (Fig. 2). The transverse colon was resected ~15 cm (5.91 inches) proximal to the splenic flexure and 6 cm (2.36 inches) proximal to the ileocecal valve. A stapled side-to-side functional end-to-end anastomosis was created. The resected colonic specimen measured 80 cm (2.62 ft) in length, and 95 cm (8.62 ft) when stretched (Fig. 3). The patient was discharged 3 days later upon regaining bowel function. Six months later, the patient reported improved quality of life with minimal constipation, abdominal discomfort, and bloating.

## Discussion

Objective measurements including colon transit time and total fecal loading assist in the classification of chronic constipation. Previous studies have indicated that increased colon transit time is more common in women and patients with functional bowel disorders and is positively correlated with segmental and total fecal loading. Women are also more likely to have an increased mean length of the colon, leading to more frequent complications related to dolichocolon [9]. Furthermore, those who underwent operations for increased fecal load and colon transit time were more likely to have dolichocolon and were found to have higher rates of appendectomy [2].

There are diverse etiologies of abdominal pain and constipation in the pediatric patient population, ranging from congenital anomalies and infectious etiologies to functional disorders. Reports of appendicitis mimicry have been documented in literature, and only rarely are accurately attributed to dolichocolon. There have been rare cases documenting dolichocolon with associated volvulus, megacolon, obstruction, and constipation [10, 11, 12, 13]. Dolichocolon could be linked to chronic constipation, intestinal obstruction, and other mass-effect complications including hydronephrosis in pediatric and young adult populations [5, 12].

The management of slow transit constipation with severe manifestations can be difficult to manage with medical therapies and typically require surgical intervention for resolution. As colonic redundancy is the most common cause of slow transit constipation, subtotal colectomy in patients with dolichocolon can lead to improvement in constipation, quality of life, and constipation-related complications [7, 8].

Current criteria for dolichocolon diagnosis include clinical and diagnostic domains. Clinically, a patient must have abdominal pain, distension, and constipation. Diagnostic findings include (i) sigmoid loop rising over the line between iliac crests, (ii) transverse colon below the line between iliac crests, and (iii) extra loops at the hepatic and splenic flexures [3]. There are no published criteria for cellular confirmational diagnosis of dolichocolon. However, recent data supports cellular evidence of late-stage dolichocolon described as morphologic atrophy and sclerosis of the colonic neuromuscular-apparatus [1]. There are no standard algorithms for dolichocolon management at this time. However, based on the early onset clinical, radiographic, and objective measurements of dolichocolon, clinicians should have a high suspicion for dolichocolon to provide accurate and appropriate intervention in a timely manner, to which future algorithms must be generated to support this cause [14, 15].

### Presentation

*HCA Texas Interdivisional 3rd Annual Research Symposium*: 19 May 2023. *HCA Central West Texas Division 45th Annual Research Symposium*: 21 April 2023.

## Financial disclosure

All authors have no financial or personal relationships relevant to this article to disclose.

## Conflict of interest statement

None declared.

## Funding

This research was supported (in whole or in part) by HCA Healthcare and/or an HCA Healthcare affiliated entity. The views expressed in this publication represent those of the author(s) and do not necessarily represent the official views of HCA Healthcare or any of its affiliated entities.

## Previous publications

This article will be presented as an oral presentation at the *HCA 4th Annual CWTX Divisional Research Symposium* on 21 April 2023 and at the *HCA 4th Annual Texas Inter-Divisional Research Symposium* on 19 May 2023.

## Data availability

The authors confirm that the data supporting the findings of this study are available within the article and its supplementary materials.
